# Bio-actuated microvalve in microfluidics using sensing and actuating function of *Mimosa pudica*

**DOI:** 10.1038/s41598-022-11637-3

**Published:** 2022-05-23

**Authors:** Yusufu Aishan, Shun-ichi Funano, Asako Sato, Yuri Ito, Nobutoshi Ota, Yaxiaer Yalikun, Yo Tanaka

**Affiliations:** 1 Laboratory for Integrated Biodevice, Center for Biosystems Dynamics Research (BDR), RIKEN, 1-3 Yamadaoka, Suita, Osaka 565-0871 Japan; 2 Graduate School of Frontier Biosciences, Osaka University, Suita, Japan; 3 Graduate School of Nara Institute of Science and Technology, Ikoma, Japan

**Keywords:** Plant sciences, Actuators, Materials for devices, Sensors and biosensors

## Abstract

Bio-actuators and sensors are increasingly employed in microscale devices for numerous applications. Unlike other artificial devices actuated by living cells or tissues, here we introduce a microvalve system actuated by the stimuli-responsive action plant, *Mimosa pudica* (sleepy plant). This system realizes the control of the valve to open and close by dropping and recovering responses of *Mimosa pudica* branch upon external physical stimulations. The results showed that one matured single uncut *Mimosa pudica* branch produced average force of 15.82 ± 0.7 mN. This force was sufficient for actuating and keeping the valve open for 8.46 ± 1.33 min in a stimulation-recovering cycle of 30 min. Additionally, two separately cut *Mimosa pudica* branches were able to keep the valve open for 2.28 ± 0.63 min in a stimulating-recovering cycle of 20min. The pressure resistance and the response time of the valve were 4.2 kPa and 1.4 s, respectively. This demonstration of plant-microfluidics integration encourages exploiting more applications of microfluidic platforms that involve plant science and plant energy harvesting.

## Introduction

Actuator is a key unit for driving various movements in different systems such as walking, stretching and swimming. But conventional actuation methods involve complex assemblies, high dependency on external power supply and extra triggering mechanisms. More importantly, these actuating methods are not self-driven, depending external energy source, insufficient control in small-scale outputs and difficult to apply in delicate biological conditions. Instead, systems actuated by living biological materials are gradually attracting attention^1^ by addressing these conventional shortages. There are many living actuators reported in the past such as bacteria^2,3^, nervous cells^4^, blood vessels or tissues^5^ that produce energy or provide power source in various application^6,7^. Especially, integrating those living actuators with the microelectromechanical systems (MEMS) technology has achieved many unique on-chip applications^8–11^. In previous work, for instance, we have developed the bio-actuated microfluidic valves and pumps that are driven by earthworm muscle^12,13^, cardiomyocyte sheets^14^ and vascular muscle cells^15^. Also, other studies have reported that the use of skeletal muscle tissue^16–20^ and cardiomyocyte contractile force^5^ successfully achieved small objects transportation and fluid control in microfluidics.

However, there are a number of limitations to apply these living actuators in practical applications. For instance, these living actuators such as neurons, blood vessels or intestines require strict environmental control, delicate handling and complicated preparation procedures. Furthermore, ethical restrictions remain as a critical issue for their applications. Therefore, living actuators free from these concerns are highly preferred.

On the other hand, there are approximately 320,000 species of plants^21^ in the world that have been our main source of food, medicines, and clothes. Plants constantly produce energy to grow mainly from sunlight via photosynthesis function. Additionally, plants even can remain alive even if they are cut into slices as long as necessary elements such as water, air, and light are provided. More importantly, plant-related experiments are less concerning from ethical issues^22^. Although most plants are commonly considered as non-motile organisms, some plants have developed a special ability to sense the environmental stimulations and respond with prompt reactions.

*Mimosa pudica* is one of such typical stimuli-responsive plants, so called “action plant”, that rapidly shrink leaves and drop branches by responding to various external stimulations such as light, temperature, mechanical forces or attacks by insects and pathogens. Recovery and reaction against the stimulation of *Mimosa pudica* is demonstrated in the Supplementary Video S1. This stimuli-responsive behavior of *Mimosa pudica* have been widely studied from various fields including electrical^23^, mechanical^24^ and biomedical^25^ areas. When a *Mimosa pudica* branch is stimulated, the branch on the main stem drops by bending its pulvinus. The pulvinus is a joint-like bulging structure that controls inside and outside ion concentrations of plant motor cells^26^. This repetitive stimuli-responsive feature makes *Mimosa pudica* an ideal candidate for developing living actuator systems driven by the reactive stimulation. Additionally, *Mimosa pudica* are favorable to be used for device experiments from the viewpoint of highly reproductive and relatively cheap characteristics in addition to the general advantage of using plants explained above.

When considering the application of *Mimosa pudica* to the creation of specific device, a microfluidic valve can be an excellent example because a valve can work even by the relatively long cycle time of the plant actuation. Microfluidic technology is deeply involved to biology, chemistry and biomedical studies^27^. A valve is an important component in microfluidics to realize various functions such as drug delivery, biomedical assays, sample preparations and biochemical analysis^28^. But conventional microfluidic valve systems require external power supply and a control system to manipulate the flow^29^. Also, according to recent reports, microfluidics is gradually being applied to various plant-related studies such as tip-growing plant cells in microfluidics^30,31^, plant root research in microfluidics^32^ and plant-on-chip research^33^. These applications rely on the microfluidic unique advantages of precise controllability of sample volume in the plant-incorporated microfluidic platforms.

**Figure 1:**
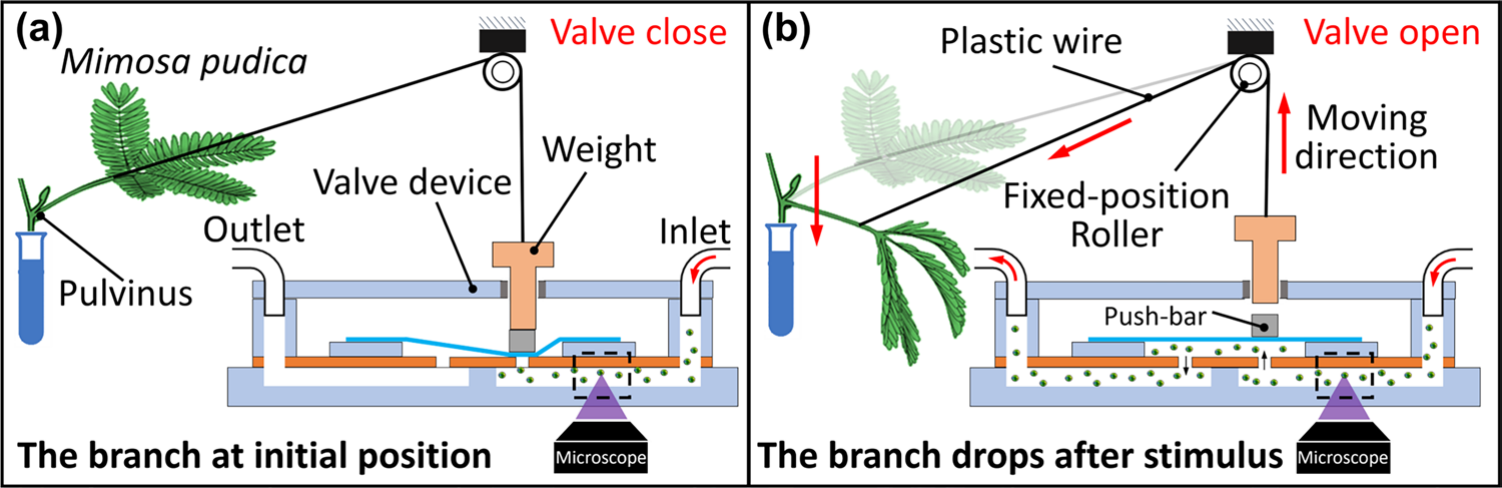
Schematic illustrations of the working principle of Mimosa pudica-actuated valve. (**a**) Before the stimulation, the weight presses down the push-bar to make the valve at the close state. (**b**) After stimulation, the branch drops and pulls up the weight, which results in connecting the inlet and the outlet microchannels. Valve changes to the open state. These illustrations are not to scale.

But, so far, most of these plant-incorporated microfluidic platforms are designed for investigating deformations, growth, and root biology of plant cells^34–36^. Unlike previous studies, the main purpose of this study is to establish a plant-actuated microfluidic valve system. Actuation of this valve system is controlled by the reaction of *Mimosa pudica* branch to the external stimulations. The motion of the branch is used to control open and close states of the valve in a microfluidic chip as shown in Fig. 1.

In this plant-actuated valve system, a stable flow is constantly applied into the valve by a pressure regulator. Before stimulation, the *Mimosa pudica* branch remains at its initial position and the weight presses down the push-bar. When a deflectable thin PDMS layer blocks one orifice, the microchannel is not connected and the valve system is at the close state. But when the branch is stimulated by mechanical contact (e.g., touching the pulvinus by a tweezer), the petiole bends downward to pull up the weight via plastic wire. At this moment, the valve switches to the open state.

## Results

### Comparison of the force exerted by *Mimosa pudica* branches in various conditions

**Figure 2:**
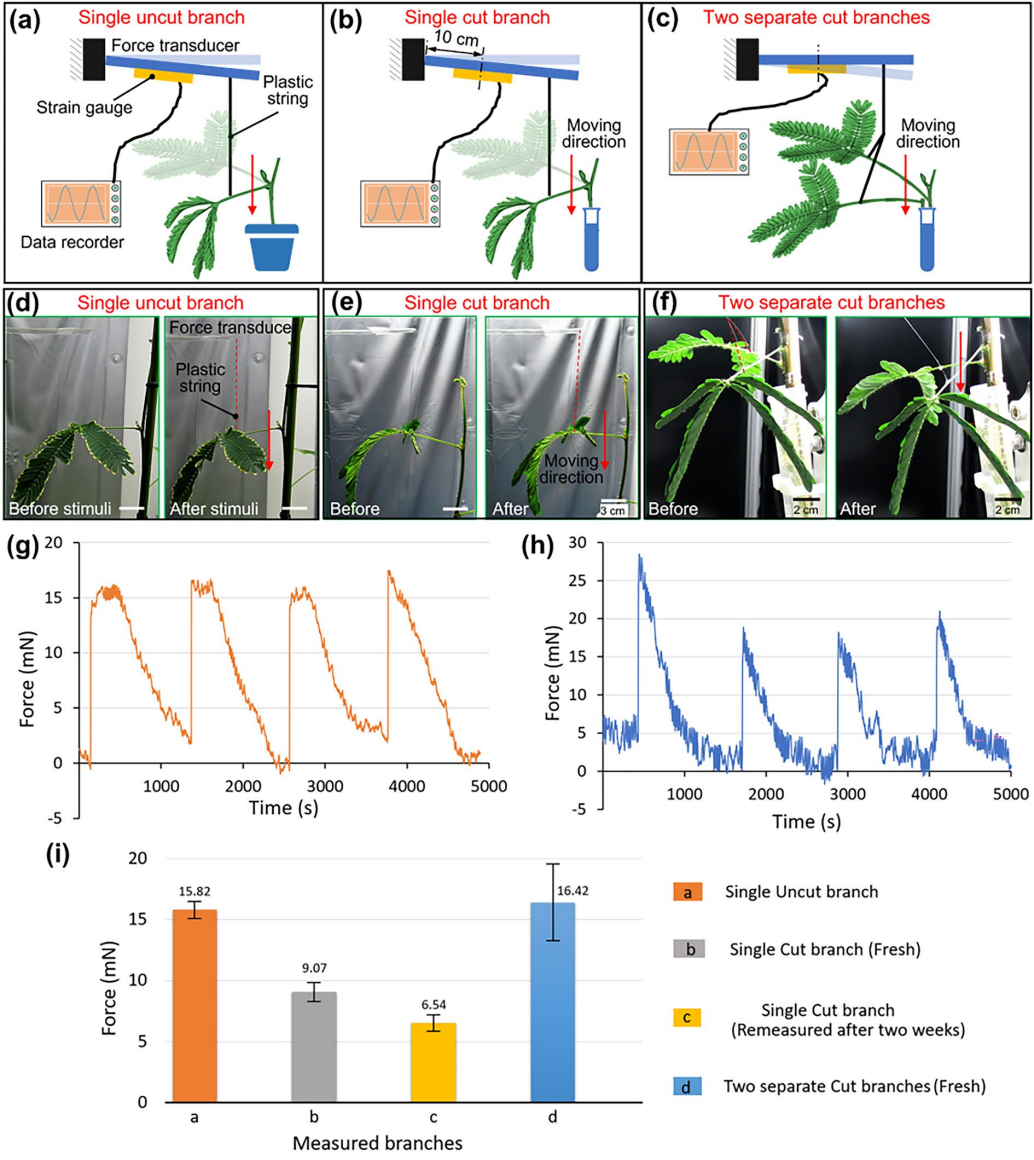
Evaluations of the force produced by *Mimosa pudica* branches. (**a**–**c**) Illustrations of the measurements for the force produced by single uncut, single fresh cut and two fresh separately cut branches, respectively. (**d**–**f**) Comparison of the different branches before and after the stimulations. (**g**,**h**) The force produced by single uncut and two fresh separately cut *Mimosa pudica* branches upon periodic stimulations. (**i**) Summary of the force produced by different types of branch samples.

To evaluate the force produced by *Mimosa pudica* upon stimulations, we measured four different types of branch samples: a single uncut branch, a fresh single cut branch, an old single cut branch and two fresh separately cut branches. For the fresh cut samples, the branches were cut 2 h before their measurements. Cut samples were immersed in tap water. For the old cut samples, we referred to the branches that were used after 2 weeks from their original cut. During the experiment, all *Mimosa pudica* branches were stimulated by touching the main pulvinus using a tweezer. Each cycle of stimulation was performed at a 30-min interval. The measurement results are shown in Fig. 2.

As shown in Fig. 2i, among single branch samples, the uncut single branch sample produced the highest stimuli-responsive force which was 15.82 ± 0.7 mN (n = 4, error means ± SD). The setups for measuring force are shown from Fig. 2a–f for different samples of *Mimosa pudica* branches. For preparation of the fresh cut branch samples, it required 2 h to setup before the measurements. Stimuli-recovery cycle of a single fresh cut branch is demonstrated in the Supplementary Video S2. In the preparation, a single branch was cut off with its stem at a 12–20 cm range of length as shown in Fig. 2b,e. The fresh single cut branch produced average force of 9.07 ± 0.76 mN (n = 4, error means ± SD). The same procedures were used to prepare the two separately cut branches and they were wired together to the cantilever for the measurement as shown in Fig. 2c,f. The single or two separate cut branches were anchored both at the bottom and on top to the tube, fixed the stem and left only the branch free for the simulation. Figure 2g,h show the force produced by the single uncut branch and two fresh separately cut branches, respectively. The two fresh branches produced average total force of 16.42 ± 3.16 mN (n = 4, error means ± SD). Each measurements were conducted in the same 20-min stimuli-recovery cycles. Also, the measurements showed that two fresh separately cut branches generated the largest force but lasted short than 4 min. On the other hand, the single uncut branch produced smaller force than the cut branches but lasted longer, typically 6 to 8 min. Both types of branches, single uncut and two fresh separately cut samples, produced sufficient force for actuating the valve device. Finally, the single *Mimosa pudica* branch was measured again after two weeks from the initial cut. It still produced average total force of 6.54 ± 0.66 mN (n = 4, error means ± SD). These results showed that the actuating force produced by stimuli-responsive behavior of *Mimosa pudica* was relatively strong than our previous studies where the earthworm muscle tissue produced the force of 1.57 mN^12^ and the smooth muscle cells (SMCs) produced the force of 1.1 μN^15^. Thus, we concluded that *Mimosa pudica* could be used as a power source for actuation to achieve open and close control of the microvalve.

### Evaluating displacements of different weights pulled by the *Mimosa pudica* branch

**Figure 3:**
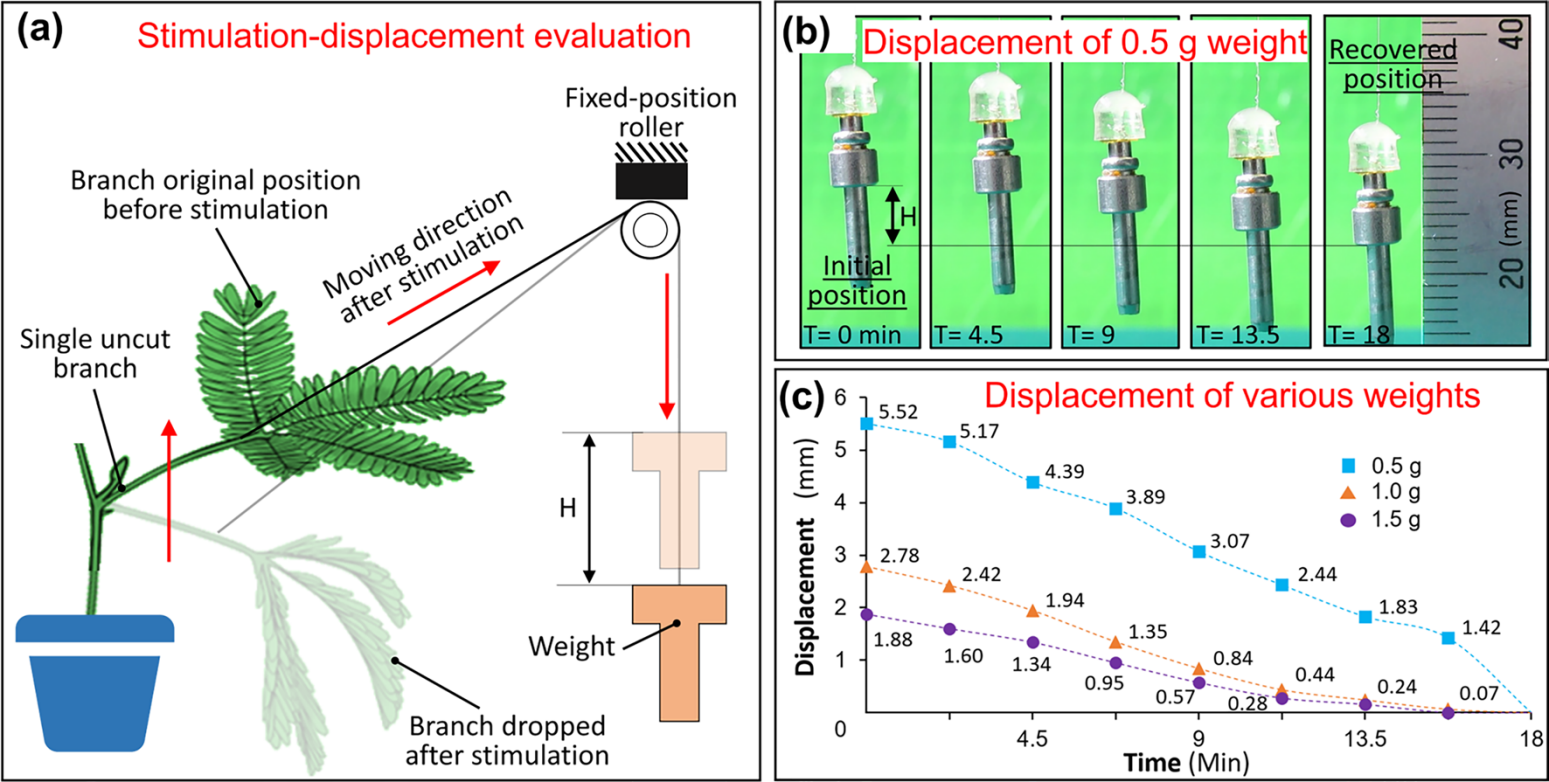
Displacement of a weight upon stimulating single uncut *Mimosa pudica* branch. (**a**) The setup for measuring the displacement. (**b**) A series of pictures of 0.5-g weight. The weight moved as the branch recovered from the stimulation. (**c**) Displacement time-course of different weights performed by the same sample of single uncut *Mimosa pudica* branch.

Figure 3a showed the setup for stimulation-displacement evaluation by using a single uncut *Mimosa pudica* branch. We measured the displacements of three different weights (0.5, 1.0, and 1.5 g) that were pulled up by stimulating the single uncut branch. Figure 3b showed the sequential images of the 0.5 g of weight’s displacements during recovery of the uncut single branch from the stimulation. As shown in Fig. 3c, the displacement of 0.5, 1.0, and 1.5 g weights were 5.52 ± 0.57 mm, 2.78 ± 0.48 mm, and 1.88 ± 0.17 mm (n = 4, error means ± SD), respectively. It was confirmed that 1.5 g were the maximum weight for the single uncut *Mimosa pudica* branch to pull. The plots represent the displacement time-course of weights from their original positions (the weight position before stimulating the branch). Time is used on the x-axis because it shows how long the weight can be lifted during the recovery process of the branch. The result shows that even using 1.5 g wight, displacement over 1 mm (required to open the valve) can be maintained for about 5 min. This demonstration is given in the Supplementary Video S3.

The minimum starting flow pressure, necessary for connecting inlet and outlet microchannels, was found to be between 4.2 to 4.5 kPa. Additionally, a minimum weight of 1.5 g was needed to stop the minimum flow. The confirming processes are demonstrated in the Supplementary Video S4. As shown in Fig. 3c, 1.5-g weight could be pulled up for nearly 2 mm, which was sufficient displacement for functioning the valve device. Hence, a single uncut branch and two fresh separately cut branches were expected to produce sufficient force to actuate the valve device.

### Demonstrations of microvalve actuation by *Mimosa pudica* branch

#### Microvalve actuation by a single uncut *Mimosa pudica* branch

**Figure 4:**
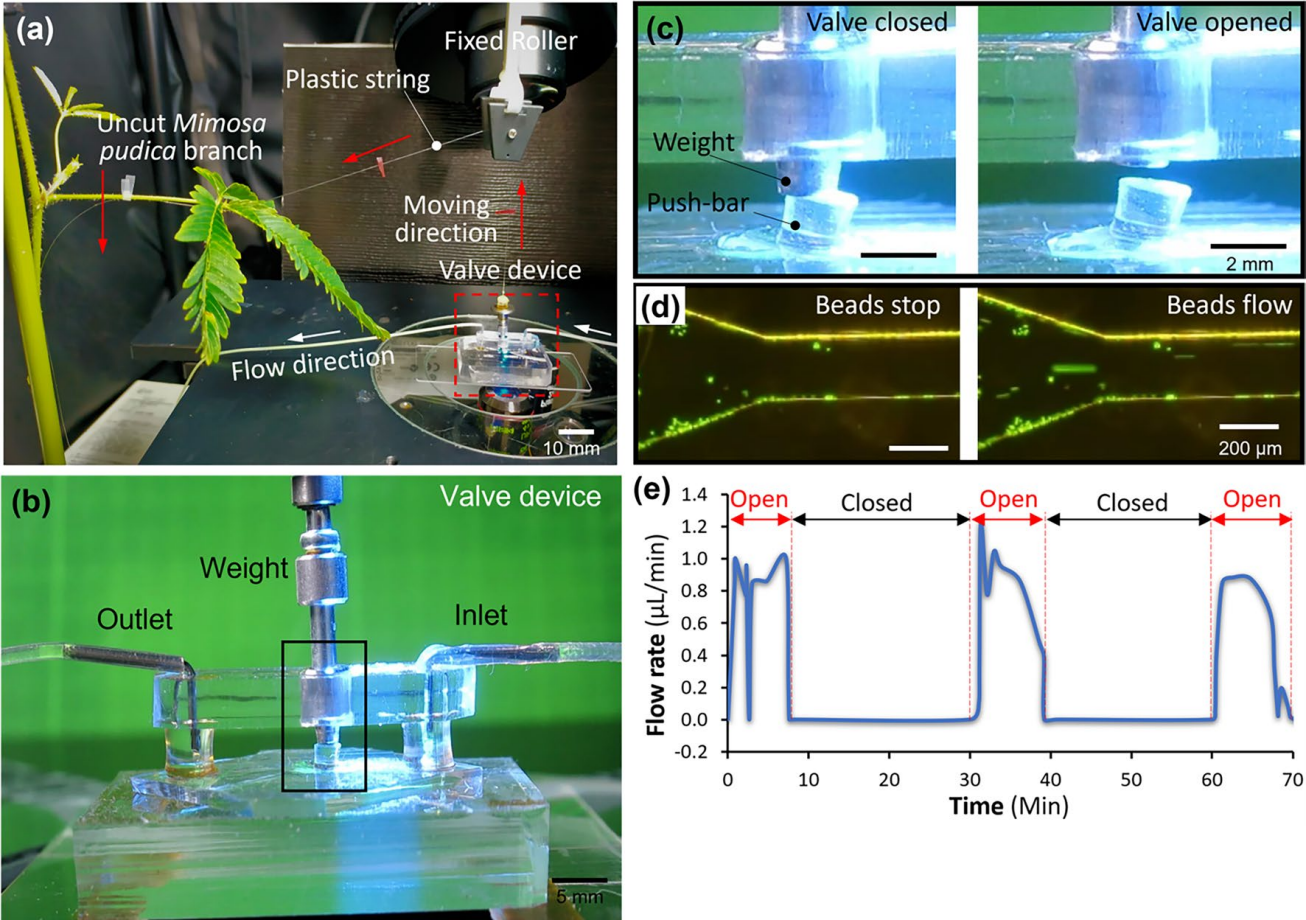
The demonstration of the microvalve actuated by a single uncut *Mimosa pudica* branch upon stimulations. (**a**) The overall setup. (**b**) A front view of the valve device. (**c**) Different positions of the weight on the push-bar before and after the stimulation. (**d**) Pictures of flowing and stopped fluid in the inlet microchannel before and after the stimulation. (**e**) Measured flow rates in the microchannel from each stimulation cycle.

Figure 4 showed the setup and results of actuating the valve device by an uncut single *Mimosa pudica* branch. A healthy *Mimosa pudica* branch was positioned near the microscope and was wired with a plastic string to the 1.5 g weight as shown in Fig. 4a.

Before stimulating the branch, the 1.5 g weight pressed the push-bar and the valve stayed at the close state. When stimulation was carried out by touching the main pulvinus, the branch dropped within 1 s and then started the recovering period for several minutes. Such dropping and recovering movements of the branch achieved open and close control of the valve as shown in Fig. 4c. A stable fixed flow was constantly applied during the stimulation.

The demonstration of the valve by single stimulation is given in Supplementary Video S5. The demonstration of multiple simulations is given in Supplementary Video S6. Each of this stimuli-recovery cycle was conducted at 30-min intervals and carried out 3 times using the same branch. The valve function was confirmed by the movement of polystyrene beads. The results showed that the time to keep the valve open in one cycle was 8.46 ± 1.33 min (n = 4, error means ± SD) as shown in Fig. 4e. From the videos, the response time of the valve using uncut branches were measured to be 1.37 ± 0.27 s (n = 3, error means ± SD).

Time to keep the valve open in the 3 cycles were 7.35 min (first cycle), 8.09 min (second cycle) and 9.94 min (third cycle). The large change of the time was not observed. This result is comparable to the measured data of direct pulling force in Fig. 2g which shows no significant difference in each cycle. The difference occurred possibly by disruptions, such as vibration of compressor for fluid control systems, compartment temperature changes due to the long-term radiation, and less humid condition.

Also, polystyrene beads were partially aggregated due to the hydrophobic effect although the flow velocity was not significantly influenced by this phenomenon because the size of the microchannel was much larger than the size of the beads. In addition, only liquid would be used in most of practical use. Hence, particle aggregation is unlikely to cause a serious issue.

#### Microvalve actuation by two fresh separately cut *Mimosa pudica* branches

**Figure 5:**
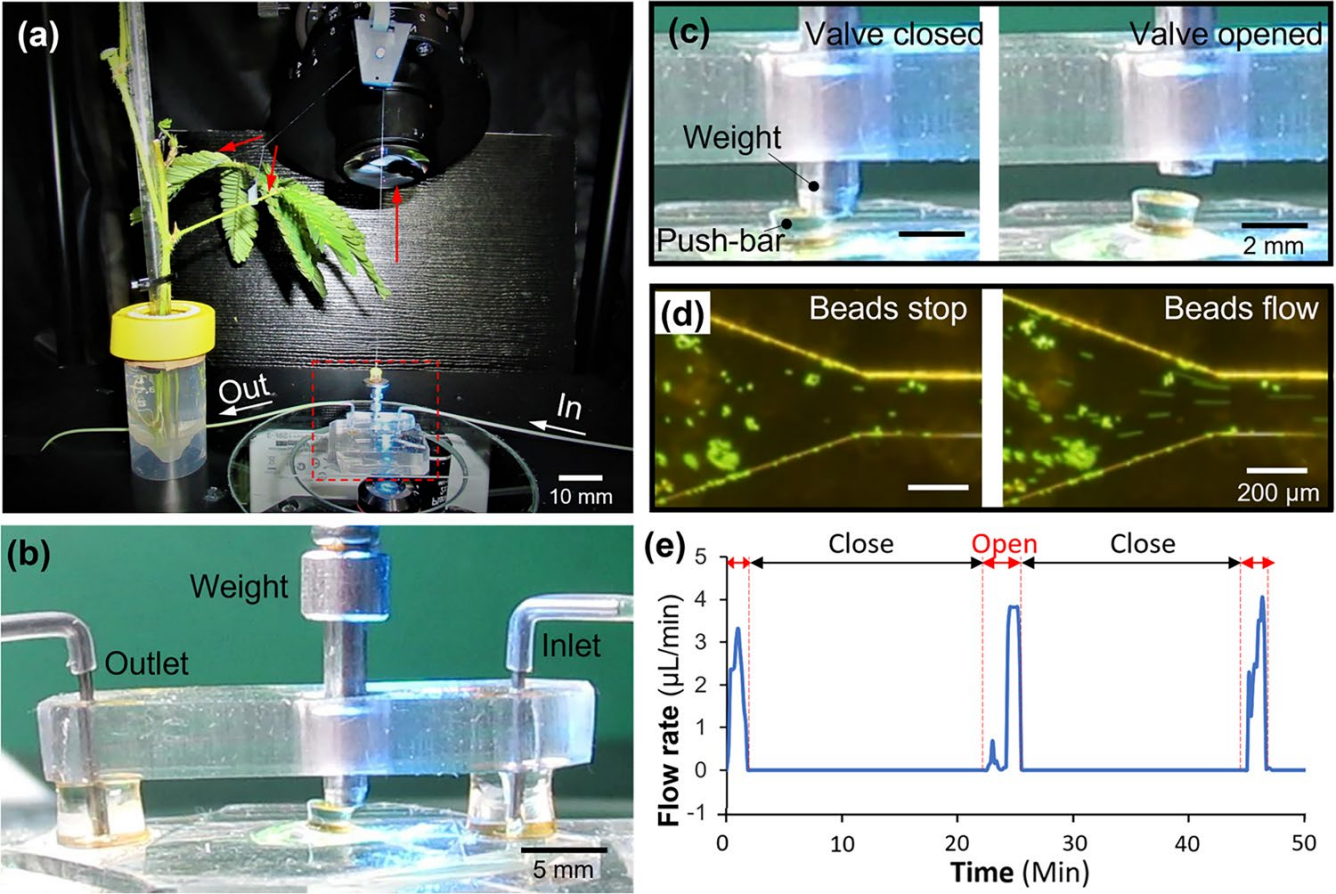
The demonstration of the microvalve actuated by two fresh separately cut *Mimosa pudica* branches. (**a**) The overall setup for actuation. (**b**) A front view of the valve device. (**c**) Different positions of the weight on the push-bar before and after the stimulation. (**d**) Pictures of flowing and stopped flow in the inlet microchannel before and after the stimulation. (**e**) Measured flow rates in the microchannel from each cycle of stimulation.

One of the unique advantages of the plant comparing to the other living substances is that plants can keep growing without losing its function as long as the proper air, water and light are constantly provided. Based on this fact, we performed the same experiment and actuating the valve device using cut branches. According to the results of the force exerted by cut *Mimosa pudica* branches, only one cut branch could not produce enough force to pull up the 1.5-g weight to achieve the valve functions. This valve's overall synchronicity is achievable because the necessary weight for opening or closing the valve is determined. A single cut branch-produced force is not enough to activate the valve, so the "quick" response branch always waits for the "slow" branch joining effect to activate the valve. Therefore, we employed two fresh separately cut branches to conduct the experiment of actuating the valve, as shown in Fig. 5. All parameters and conditions were the same with the earlier demonstration performed with single uncut branch.

Figure 5a shows the overall setup for microvalve actuation by two fresh separately cut *Mimosa pudica* branches. The demonstration of the valve is given in Supplementary Video S7. Each cycle of this experiment was conducted in 20-min intervals and carried out 3 times using the same branches. The valve function was confirmed by the movement of polystyrene beads. The results showed that the time to keep the valve open in one cycle was 2.28 ± 0.63 min (n = 3, error means ± SD) as shown in Fig. 5e. It was found that the actuation produced by two fresh separately cut branches could keep a shorter period of opening valve compared to those produced by the actuation of single uncut branch. This is comparable to the data shown in Fig. 2. From the videos, the response time of the valve using two cut branches were measured to be 1.75 ± 0.35 s (n = 2, error means ± SD).

Regarding how long the valve can keep fluid controlling function after multiple cycles, we could confirm the functionality at least 3 h before the experiment finished. Moreover, as shown in Fig. 2, a branch after two weeks from cutting could keep the actuating function. So, potentially the valve function can be maintained for such a long period (more than 2 weeks). In other words, if the temperature, water and nutrition are properly provided, the cut branches can be kept alive and grow to keep the valve functional. There are also additional information on the single cut branch evaluations in the Supplementary Figs. S1 and S2.

## Discussion

Here, we compared our valve device actuated by *Mimosa pudica* with previously reported valve devices involving microfluidic systems. We have compared the various valve devices based on their control methods, drivers, pressure resistance and response time. The summarized results are given in Table 1.

**Table 1:** Comparison of various active valves based on control methods of actuation, pressure regulations and response time (n/r denotes not reported).

Control methods	Driver	Regulating pressure (kPa)	Response time (s)	References
Physically stimulating	Plant motor cells (ion concentration changes)	4.2	1.4	This study
Electrical	Piezo (bimorph)	3	0.1	^37^
	Earthworm	3	3	^13^
Chemical	pH responsive hydrogel	39	n/r	^38^
	pH responsive hydrogel	n/r	8	^39^
	Earthworm	15	42	^13^
Magnetic	Permanent magnet	200	0.05	^40^
	PDMS-iron	n/r	n/r	^41^
Optical	Photo-responsive polymer gels	10	30	^42^
	Poly(*N*-isopropylamide)	n/r	5	^43^
Thermal	Polyurethane	200	10	^44^
	PDMS-silver	200	23	^45^
	Polyethylene glycol	10	60	^46^
	Thermo-responsive fluid	10	7–80	^47^
Pneumatic	Air	60	0.01	^48^

Generally, active valves are driven or controlled by external manipulations such as electrical^13,37^, chemical^38,39^, magnetic^40,41^, optical^42,43^, thermal^44–47^ or pneumatic^48^ actuations. Since response time and pressure resistance of valves heavily depend on the microchannel structure and valve sizes, we have only evaluated the valves that produce relatively large displacement (~ 100 μm). Comparing our valve driven by *Mimosa pudica* with conventional valves with quick response (within 1 s) such as electrically, pneumatically, or magnetically actuated valves, the *Mimosa pudica*-driven valve does not show quick response to the outer stimulations (1.4 s) and the valve required the period of recovery which took a relatively long time, approximately 15 min.

However, the *Mimosa pudica* valve is not always slow when compared with chemically, optically or thermally controlled polymer or hydrogel^39^ ones which show slow volume change and low sensitivity to the stimulations. Also, our earthworm muscle-driven valve^13^ took 42 s to respond to the stimulation by acetylcholine solution. The biggest disadvantage of this earthworm muscle-driven valve is the actuating drivers composed of animal cells which cannot constantly work for a long time, significantly lose its functionality within an hour, and difficult to recover.

The response time and the pressure resistance of the *Mimosa pudica*-driven valve are at comparable level compared with previously reported on-chip valves used in microfluidics.

Also, a wide range of triggering options are available for this plant valve combination for various applications such as touching, sudden shock, strong wind, raindrops, light radiation, temperature and humidity of the environment^49^, etc. To reduce the accidental or the multiple overlaps activation, certain chemicals such as ether, acetylene, chloroform, and ethylene can be used to manipulate the plant's response level or sensitivity to the stimulations^50,51^. In this study, we only considered the single trigging condition.

The optimization is necessary through miniaturization of devices. For miniaturization purposes, it is important to know how much the actuating components of *Mimosa pudica* can be made smaller because it is a principal component of this valve. Therefore, we have checked the feasibility of using immature branches (ones obtained after 3 weeks from seeding) that are smaller than mature branches (ones obtained after more than 2 months from seeding). We compared the size and weight of a mature cut branch that was used in the experiment with an immature cut branch, as shown in Supplementary Fig. S3. The size (diameter from top view) and weight of the mature branch were about 10 cm and 0.9 g. On the other hand, the size and weight of the immature branch were about 5 mm and 0.02 g. Even when using the immature branch, it was actuated by the physical stimulation as shown in Supplementary Video S8. Even though the force was difficult to measure in this scale and the displacement was small, it would be sufficient to be used for a valve because the valve requires only a few hundred micrometers of displacement. From this result, the possibility was indicated to miniaturize a device to 5% or less of the current device.

Not only actuators (plants) but also design and fabrication method must be considered to construct effective structure to convey the plant-generated force to the fluid for actual miniaturization. For example, sheet-like structure^12^ and dome-like structure^52^ using leaves or integration of leaf pillow (expanding and contracting parts of *Mimosa pudica* at the root of the branches) into a microchannel like hydrogel-based valves^38,39,42,43^ are the candidates for realizing such integrated valves in a small space. Fabrication of devices using more flexible materials such as hydrogel or gelatin^53,54^ would also be required.

As demonstrated in Supplementary Fig. S3 and Supplementary Video S8, the valves can be miniaturized to be smaller than 5 mm which is smaller than the microchip in this study (3.5 cm × 2.0 cm). Therefore, multiple valves can be incorporated into a single chip and each valving point can be controlled by individual branches just by simple touching to operate the direction of flow in the chip. That is similar to the operation of a smartphone only by touching its screen. Usually, microvalves used in microfluidic systems are bulky if controller and power source are included^55^. In case of using a compressor for operating pneumatic valves^48^, noise and vibration are also inevitable. Compared to these conventional microvalves, the plant-based valve is free from these issues. Therefore, the smartphone-like on-chip fluid controlling device would be a suitable application for on-site usage of microfluidic systems. In addition, since this valve system is stimuli-responsive to its surrounding environment (temperature, light, humidity, etc.), it can be used for applications regulated by valves with the ability of self-adjustable flow control. Although further optimization is necessary, such unique applications would be realized based on the characteristics of this valve in future.

Therefore, the *Mimosa pudica*-driven valve has potential to develop new microfluidic applications via customized modifications. In terms of actuating force, most of the valves listed above originally rely on electricity or chemical reactions influenced by pH of the solutions and these harsh driving environments are not preferred in bio-related applications. Differently from conventional valves, the *Mimosa pudica*-actuated valve produces driving force by the changes of ion concentrations in the plant cells which has completely no harm or affect to the substances in microchannels or to the device itself.

Therefore, this *Mimosa pudica*-actuated valve can be considered as a bio-friendly valve and produces a relatively large driving force compared to the valves in our previous studies^12,14,15^. These valves in series integration with microfluidics can be used for slow and stable processing required chemical and biological analysis.

Also, it has the potentials to create an environment sensing valve and wireless sensor network for greenhouse plant monitoring, smart watering, and spray system establishment. More importantly, this study works as a fundamental demonstration using plant energy as the driving source in microfluidic devices, which paves the way for more combination of plants-microfluidics applications.

## Conclusion

In this work, we have demonstrated a microvalve system driven by the stimuli-responsive force of *Mimosa pudica*. The results confirmed that such stimuli-responsive reactions of *Mimosa pudica* branches can be used as a powering source and can be integrated with a microfluidic valve.

In the first demonstration, we used a single uncut branch to actuate the valve. The uncut branch reacted to external physical stimulations within 1 s and required several minutes for recovery to respond to the next trigger. This force was sufficient for actuating and keeping the valve open for 8.46 ± 1.33 min in a stimulation-recovering cycle of 30 min. Additionally, two separately cut *Mimosa pudica* branches were able to keep the valve open for 2.28 ± 0.63 min in a stimulating-recovering cycle of 20 min. The pressure resistance and the response time of the valve were 4.2 kPa and 1.4 s, respectively.

This study can be considered as the first demonstration of harvesting plant energy to actuating a valve device and integration of a plant-based actuator with a microfluidic system. Due to the advantages of plants including swelling/bending properties, self-healing capabilities, stimuli-responsiveness, and biocompatibility, plant-based devices appeals for various modern sciences and technologies that related to new energy exploration, environment sensor, and smart devices as well as special valves and bio-communication devices.

## Methods

### Device fabrication

**Figure 6:**
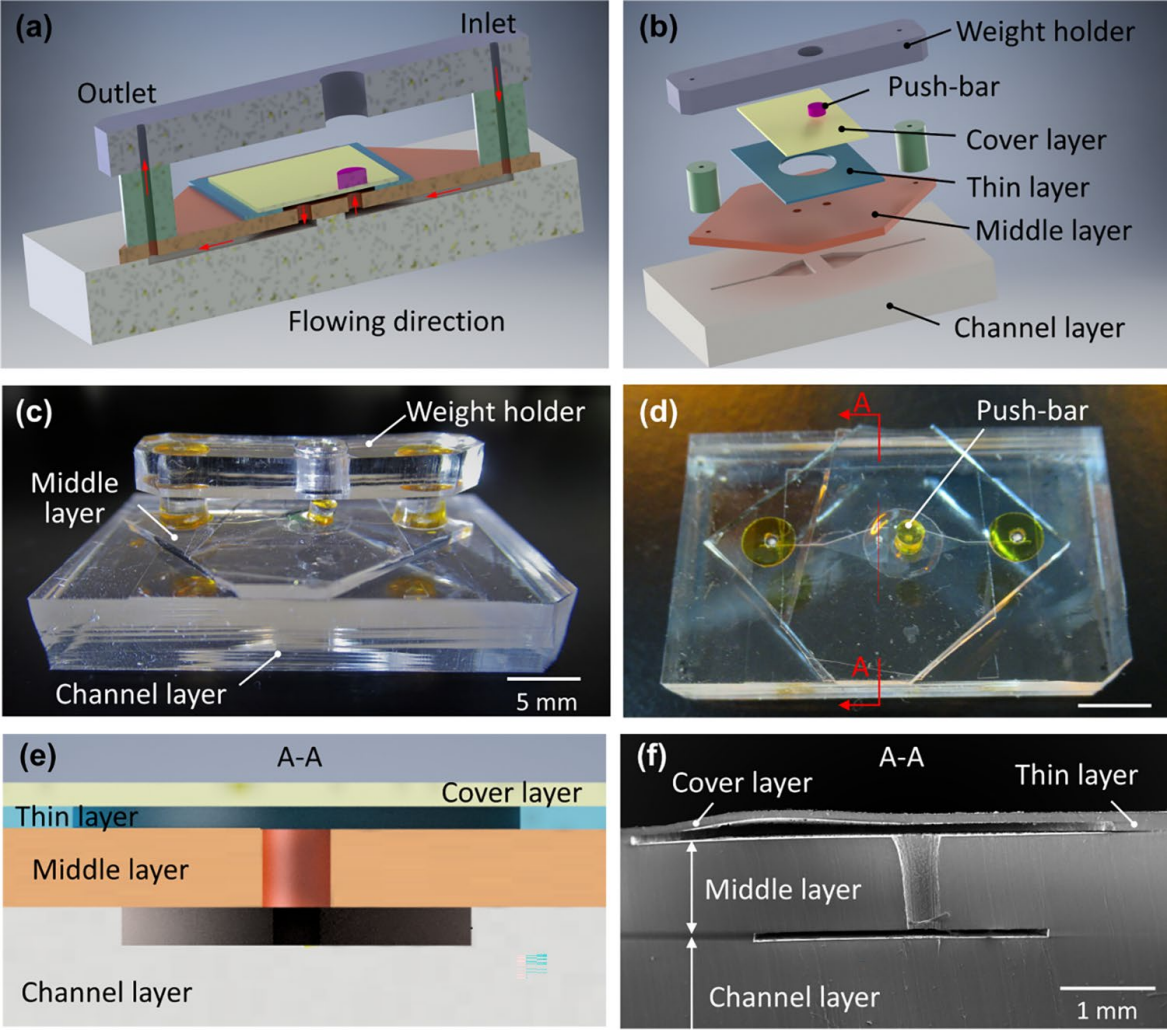
Assembly of the valve device. (**a**) A cross-section of the valve device. (**b**) The main parts of the valve device. (**c**) The side view of the assembled valve device. (**d**) The top view of the valve without the weight holder. (**e**) A conceptual cross-sectional illustration of the valve. (**f**) The SEM cross-sectional image of the valve.

Fabricated valve device is shown in Fig. 6. This device is made of three parts: the weight holder, the push-bar, and the valve body. First, the weight holder is the top part of the valve device. Its function is to guide the weight to move in vertical directions. Second, the push-bar in the middle is for transmitting the force produced by *Mimosa pudica* from the weight to the deflectable thin PDMS layer. Third, the valve body is composed of three PDMS layers which were bonded together by oxygen plasma as shown in Fig. 6a. The microchannels were imprinted on the channel layer in the valve body through the replica molding method using a silicon wafer. The PDMS prepolymer (Silpot 184 W/C, Dow Corning Toray, Tokyo, Japan) and photoresist (SU-8 3050, Nihon Kayaku, Tokyo, Japan) were used for the fabrication of the microfluidic device.

The width of both the inlet and the outlet microchannels started at 200 µm and widened up to 3 mm at the end as shown in Fig. 6b. The depth of the microchannels was approximately 100 µm. The inlet and the outlet channels were connected via two orifices of 500-µm diameter on the middle layer. As shown in Fig. 6b, a diameter 6-mm of chamber was fabricated on the thin layer of 100-µm thickness by a biopsy punch. This chamber was covered by a cover layer of 100-µm thickness. Then, oxygen plasma was applied to bond the thin layer with the cover layer. The cross-sectional image (Fig. 6f) was captured with a scanning electron microscope (SEM) (VE-8800, Keyence, Osaka, Japan). The push-bar is a cylindrical PDMS piece of 2-mm diameter and 1-mm height. This PDMS push-bar is fixed on the cover layer using double-sided adhesive tape. There are several different points between the present device from our previously fabricated ones^56^. First, only one orifice was controlled in the present device to manipulate open and close states of the valve easily. Second, the push bar size was reduced to the half of the previous ones. This modification allows to use smaller minimum flow which increased the sensitive of the present device. Third, extra components such as holding parts and fixed-position roller were added to increase the efficiency of transmitting the force produced by *Mimosa pudica* to the valve device. Except the metal ring in the middle on the weight holder, the rest of the valve components were made of PDMS, and the valve was rectangular shape of approximately 3.5 cm × 2.0 cm.

For assembling the valve device, oxygen plasma bonding was performed in following orders. First, the cover layer was bonded to the thin layer. Then, this bonded layer was bonding with the middle layer. Finally, the channel layer was bonding with previously bonded three-layers piece. The vacuum oxygen plasma was set at 15 W for 30 s at oxygen flow rate of 8 mL/min in the chamber of a compact etcher (FA-1, SAMCO, Kyoto, Japan). Except the weight holder, the rest of the PDMS components were aligned and stacked for plasma bonding. Finally, these PDMS components treated by oxygen plasma were placed on a hot plate at 96 °C for 20 min for permanent bonding.

### Plant materials and growing conditions

*Mimosa pudica* in this experiment was wild type and had not been genetically modified or specially treated. Plant use strictly followed institutional guidelines and governmental regulations. The *Mimosa pudica* seeds were purchased from Sakata Seed (Product number 00209545, Kanagawa, Japan). This plant specimen has not been deposited in any public herbarium although this is not an important factor for this study; the present device utilized widely known sensing and actuation functions of this commercially available plant, not based on specific genetic traits, modified genes, or special phenotypes of the plant.

*Mimosa pudica* seeds immersed in warm water (25–30 °C) for 5 min before sowing them into the pots. Seedlings were cultivated in a growing compartment wrapped by temperature-controlled gray cloth. The light and dark cycle was set at 16 and 8 h. Luminous radiance was found 12.28 klx in the middle area of light source. The temperature of the growing compartment was controlled between the maximum daytime temperature of 25.1 °C and the minimum night-time temperature of 19.4 °C. The humidity was controlled in the range of 55–70% and the plants were watered every day. The plants were supplied with supplemental fertilizers until they were transferred to growing medium (Fast acting fertilizer, Hyponex Solution, Osaka, Japan). All experiments were performed on three to four-month-old healthy adult specimens.

### The force measurements of *Mimosa pudica* branch

Measurements were performed on the contractile (pulling) force produced by a branch of *Mimosa pudica* upon stimulations. The measuring system included a cantilever coupled with a strain gauge (KFW-5-120-C1- 11, Kyowa, Chofu, Japan) which was placed 10 cm away from the root of the cantilever. The strain gauge measured voltage changes that indicates the pulling force of the branch. A data logger (NR-600, Keyence) with a high-speed analog measurement system (NR-HA08, Keyence) and software (WAVE LOGGER PRO (R4.02.00 version), Keyence) were used for recording the data at 1 ms time resolution. All experiments were carried out at room temperature.

### Pressure regulation and flow observation

A constant flow was applied into the valve device which was controlled by a microfluidic flow regulator (MFCS, Fluigent, Le Kremlin Bicetre, France). Perfluoroalkoxy alkane (PFA) pipe of 0.4-mm inner diameter (IWASE, Kanagawa, Japan) was used to connect the valve with the reservoir. The constant flow contained suspension of fluorescent spherical polystyrene particles (Fluoro Spheres, 2 µm diameter, Molecular Probes, Thermo Fisher Scientific, Waltham, MA, USA) were diluted 100 folds in deionized water as in previous study^53^. For observations, we used a fluorescence microscope (IX-71, Olympus, Tokyo, Japan) equipped with an objective lens (10×, 0.30-NA), a charge-coupled device (CCD) camera (24-bit, RGB color, DP72, Olympus, Tokyo, Japan) and bandpass (460–495 nm)/long pass (510 nm cut-on) filters for excitation/emission wavelengths. All images were captured using software (cellSens XV imaging version 3.24, Olympus, Tokyo, Japan) through the CCD camera.

### Image analysis

The acquired videos and images were edited by commercial software (PowerDirector 16 (16.0 version), CyberLink, New Taipei City, Taiwan). The videos and images were analyzed by using Python (3.8.3 version)^57^ and ImageJ (1.8.0_172 version)^58^ open-source software.

## Supplementary Information

Supplementary Information.

Supplementary Video 1.

Supplementary Video 2.

Supplementary Video 3.

Supplementary Video 4.

Supplementary Video 5.

Supplementary Video 6.

Supplementary Video 7.

Supplementary Video 8.

## Data Availability

The authors declare that all data supporting the findings of this study are available within the paper and the Supplementary Information.
