# Supplementary material for: Bio-actuated microvalve in microfluidics using sensing and actuating function of Mimosa pudica
Source: Sci Rep. 2022 May 23;12:7653. doi: 10.1038/s41598-022-11637-3 (PMC9126872; doi:10.1038/s41598-022-11637-3)
Supplement: Supplementary file 1 — Supplementary Information. [file 41598_2022_11637_MOESM1_ESM.pdf]

Supplementary Information

Bio-actuated microvalve in microfluidics using sensing and actuating function of *Mimosa pudica*

Yusufu Aishan[1,2] , Shun-ichi Funano[1], Asako Sato[1], Yuri Ito[1], Nobutoshi Ota[1], Yaxiaer Yalikun[1,3] and Yo Tanaka[1,2,*]

1: Laboratory for Integrated Biodevice, Center for Biosystems Dynamics Research (BDR), RIKEN, JAPAN

2: Graduate School of Frontier Biosciences, Osaka University, JAPAN

3: Graduate School of Nara Institute of Science and Technology, JAPAN

Table of Contents:

- **Supplementary Figures and Legends (Figure S1-S3)**
- **Supplementary Movie Captions (Video S1-S8)**

*To whom correspondence should be addressed:

*E-mail: yo.tanaka@riken.jp.

TEL: +81-6-6105-5132, FAX: +81-6-6105-5132

Supplementary Figures and Legends

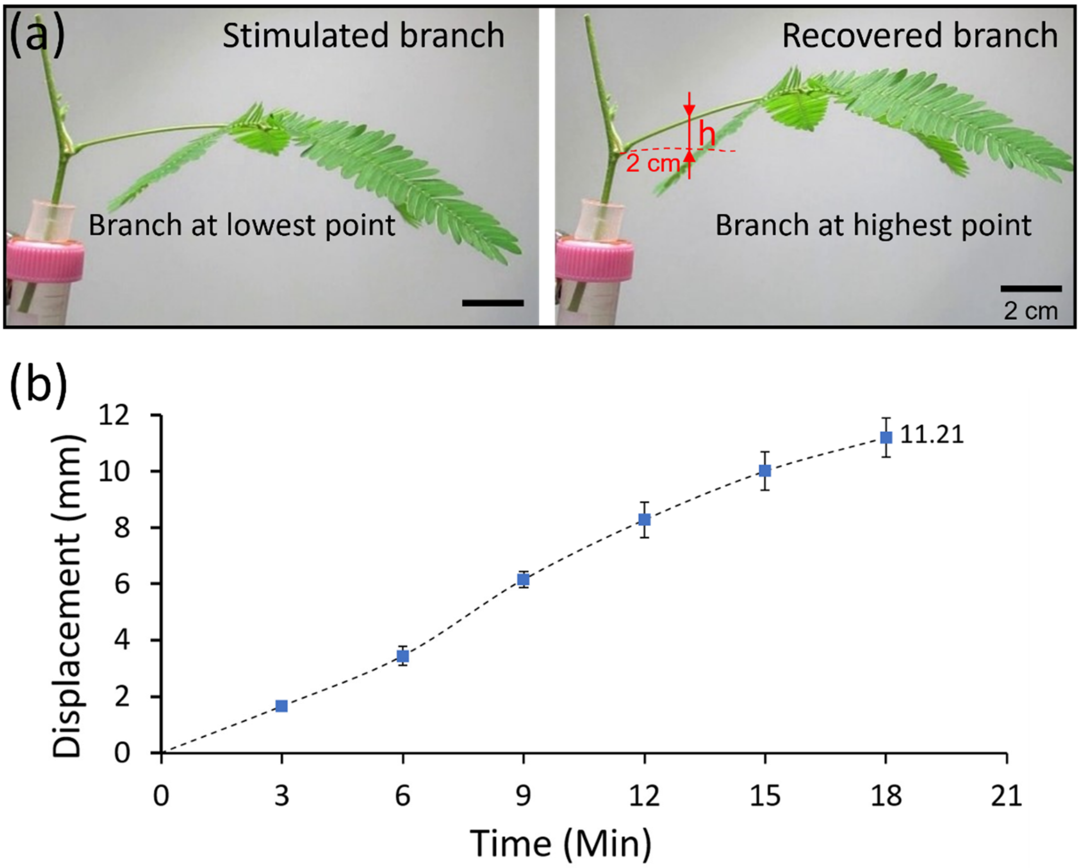

Figure S1. Displacement of a single cut *Mimosa pudica* branch during a stimuli-recovery cycle. (a) On the left, the branch is at its stimulated position by touching the primary pulvini on the stem. On the right, the branch is at its fully recovered position (its original position). (b) The measured displacement of the branch during the recovery period of a stimuli-recovery cycle. Measurements were performed at 2 cm away from the base of the primary pulvini and displacement is indicated as h in the right picture of Figure S1(a). Zero displacement was set at the lowest position of the branch found in the left picture of Figure S1(a).

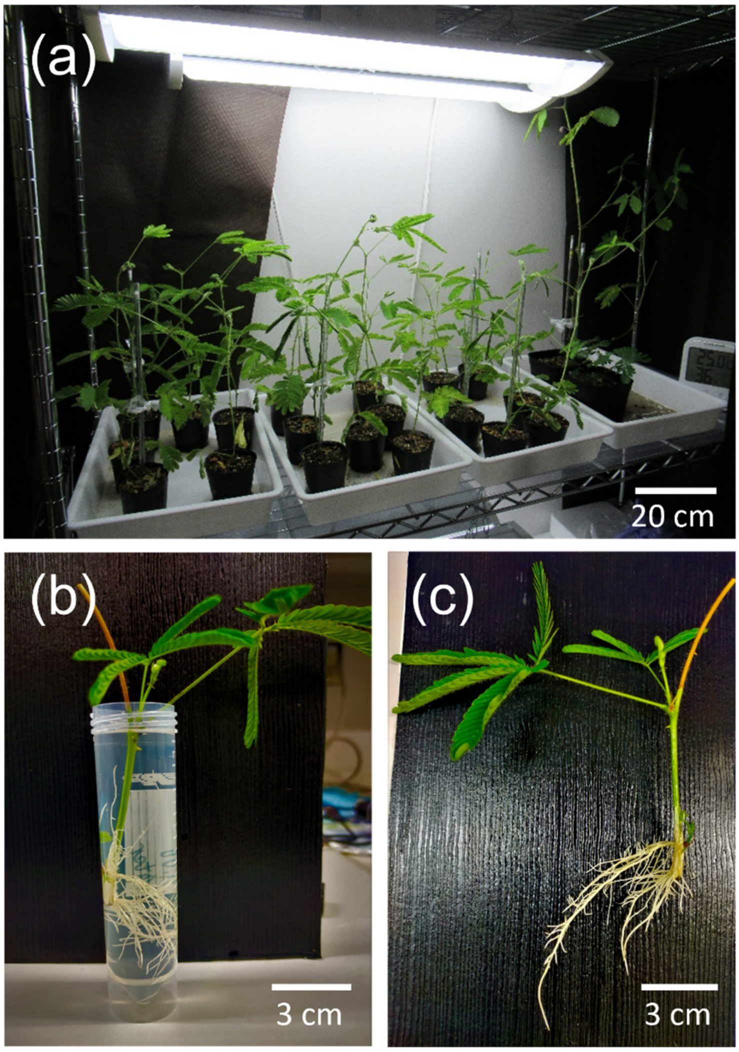

Figure S2. Growth of *Mimosa pudica*. (a) *Mimosa pudica* growing in compartments. (b) A cut *Mimosa pudica* in culture medium. (c) A cut Mimosa pudica. (b) and (c) are the same branch of a cut single *Mimosa pudica* grown in culture medium in a tube. This branch had grown for two weeks after being cut off from the stem.

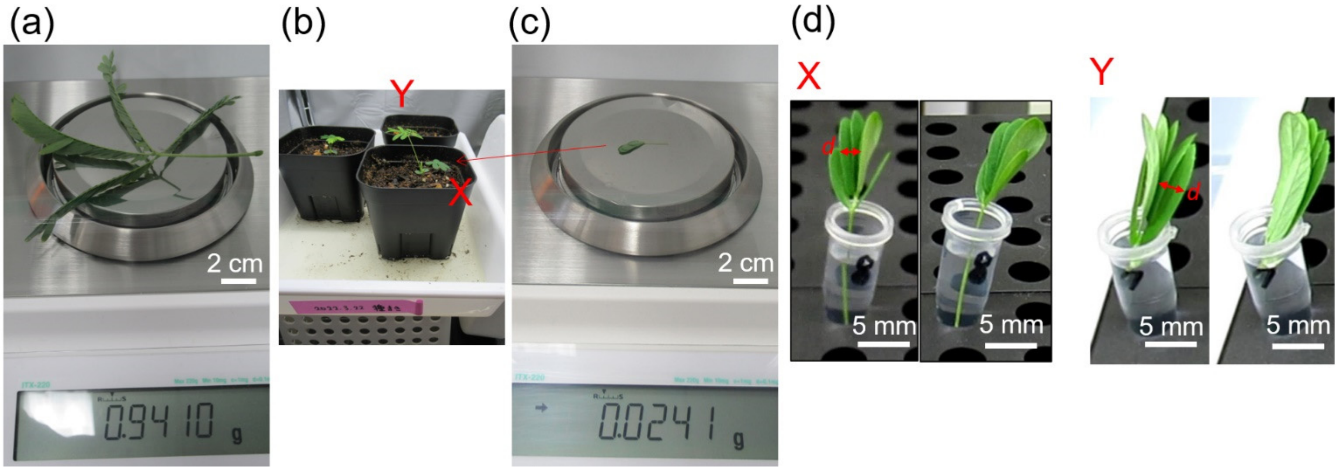

Figure S3. Comparison of the weight and the size of different *Mimosa pudica* branches. (a) Size and weight of a mature *Mimosa pudica* branch. (b) A picture of immature branches of potted *Mimosa pudica*. (c) Size and weight of an immature branch. (d) Stimuli-responsive behaviour of cut immature branches (X and Y in Figure S3(b)). Left panels show pictures before stimulation, and right panels show pictures after stimulation. Displacements upon stimulations are indicated as d with arrows in red font. Actuating motion is presented in Video S8.

Supplementary Video Legends

Video S1 | This movie shows the plant's recovery under light, that was kept in the dark overnight Also, it demonstrates the recovery of the same branch from multiple stimulations.

Video S2 | This movie shows the behavior of a single cut *Mimosa pudica* branch upon stimulations.

Video S3 | This movie shows the displacements of different weights pulled up by stimulating a single uncut *Mimosa pudica* branch.

Video S4 | This movie shows the confirmation of the minimum flow pressure to open the valve and the necessary weight to close the valve under the minimum flow pressure.

Video S5 | This movie shows the overall working principle of the valve during one complete cycle of actuation when the *Mimosa pudica* branch is stimulated.

Video S6 | This movie shows multiple actuations of a single uncut *Mimosa pudica* branch and the change of flow in the microchannel

Video S7 | This movie shows the change of flow in the microchannel by actuation of two fresh separately cut *Mimosa pudica* branches. The same chip was used in the previous experiment.

Video S8 | This movie shows the reactions of cut immature (smaller) branches to the physical touching stimulation which produce displacements.
